# Associations between Stress and Quality of Life: Differences between Owners Keeping a Living Dog or Losing a Dog by Euthanasia

**DOI:** 10.1371/journal.pone.0121081

**Published:** 2015-03-31

**Authors:** Lilian Tzivian, Michael Friger, Talma Kushnir

**Affiliations:** Department of Public Health, Faculty of Health Sciences, Ben Gurion University of Negev, Beer Sheba, Israel; Oregon State University, UNITED STATES

## Abstract

**Objectives:**

The loss of a pet may be stressful to the owner. The main objectives of this study were to compare the levels of stress and to explore the correlates of QOL of healthy adults who currently own or who have just lost their dog.

**Methods:**

The study sample contained 110 current, and 103 bereaved dog owners, all females, who lost their dogs due to euthanasia. QOL was assessed with the WHOQOL-BREF questionnaire and divided into four major domains–Physical, Psychological, Relationship, and Environmental. Demographic variables, stress, health behaviors, and social support from family, friends, and significant other were included in multivariate analysis.

**Results:**

Stress levels were significantly higher in bereaved owners. QOL in three of the four domains (Physical, Psychological, and Relationship) of current owners were significantly better than among bereaved owners. Stress was significantly associated with these three domains of QOL. Quality of life was found to be positively associated with social support. Age was related directly only to current owners’ QOL.

**Conclusions:**

The results suggest that a loss of a dog is associated with stress for the bereaved owner and reduced physical, psychological, and relationship QOL. Lack of social support in the case of death of a companion animal has a strong effect on owners’ grief reactions.

## Introduction

The self-reported physical and psychological benefits of owning a dog have been documented in social science research [1]. The research suggests that pets can provide a range of benefits to humans, including health benefits [2–4]. Since the 1980s, numerous publications have demonstrated an improvement in people’s quality of life (QOL) due to their contact with pets. The psychological benefits of pet-human interaction have been investigated mainly among sick individuals [5]. For example, a significant difference on the well-being scale was found between sick pet owners and sick non-owners [6]. While there are several hypotheses that attempt to explain the health benefits of dog ownership by focusing mainly on the nature of the relationship between owners and their dogs [7], there seems to be no agreement as to the underlying mechanisms responsible for these effects [1].

Because cats and dogs have average lifespans of about 10–15 years, much shorter than that of their owners, pet owners frequently face pet loss. The loss of a pet may be very stressful to the owner [8–11], whether these deaths were caused by euthanasia, accident, or by the progress of illness [12]. The longer one has a pet, the greater the attachment can become [13–14], and this strong attachment may provoke a distress reaction upon a pet’s death. Many people grieve for their pets in much the same way as they do for the deaths of their friends [15]. Families are known to experience a range of emotions in response on the loss of animal, such as a deep sense of sadness, grieving, crying, and even depression [16–17]. In extreme cases, the mourning resulted in hospitalization for psychiatric treatment [18], although the percentage of people expressing major pathological reactions is relatively low at 5–12% [16].

One of the most important factors contributing to the intensity of stress reactions following the loss of a pet is the owner’s age [19]. Many studies have shown that the loss of a pet resulted in a very difficult and painful time for children who felt guilty about the death and could not accept it [20]. Planchon and Templer (1996) found a more intense grief reaction among younger rather than older pet owners [21]. The relationships of adolescents with pets tend to be more intense, which may explain why it takes them longer to get over the grief [20].

Another important factor that can affect or moderate the levels of stress and well-being at the time of stressful events is social support that a person receives. Although the significant role of social support in the case of the death of a close relative has been demonstrated [22], the death of a family pet is not fully recognized as a significant loss, especially among those who do not own pets, and grieving owners are often left without strong social support [23]. Even veterinarians can underestimate the impact of the loss of a pet on their clients [24]. Such lack of social support may increase the likelihood that the bereaved person will avoid the processing of loss [25].

The main objectives of this study were to explore the correlates of quality of life of healthy adults who currently own a live dog in comparison to owners who have just lost their pet, and to compare the levels of QOL of bereaved dog owners and current owners, defined as people raising and caring for a dog for at least two years.

## Materials and Methods

### Study population

Female participants were recruited from central and southern Israel. Participants had to be able to read the questionnaires in Hebrew and respond to them without assistance. We excluded persons with disabilities, as the innovative focus of this study is healthy adults, and we therefore also excluded those who defined themselves as ill. Participants represent all types of family status (single, married, divorced, widowed), with or without children, living in urban or rural settings, alone or with their families. In addition, the participants varied greatly in their years of education and employment situation. Inclusion and exclusion criteria for groups of comparison are explained below.

#### Bereaved owners

As it is impossible find all persons who have lost their dogs, we included only those for whom we could ascertain the date of the dogs' death. This was done by checking with the veterinarians who had treated the animals and were present at the time of death. All the veterinarians in the center and south of Israel were visited personally by the researcher and asked to assist in locating owners of euthanized dogs and to obtain their consent to participate in the research. Veterinarians from 48 small private clinics were involved in this study.

Those veterinarians who agreed were asked to explain to the owners the aims of the study and to obtain their consent to participate. We contacted the veterinarians who agreed to assist weekly, to obtain phone numbers of dog owners who had euthanized their dogs during the past week and had consented to participate in the study. Owners who agreed to participate were contacted by the researcher. Owners were interviewed at home, no later than four weeks after the euthanasia.

The inclusion criteria for this group were healthy owners who had euthanized a dog—for any reason—within two to four weeks prior to the interview. All clients of veterinary clinics, who agreed to participate in the study after the death of their dog, were included. The exclusion criteria for this group were loss of communication with the owner for any reason and taking on a new dog in the period since the dog's death, before the researcher’s visit.

#### Current owners

For this group we contacted owners of live dogs who had owned their dogs for more than two years. We found several participants by using personal contacts; additional participants were located with the help of the veterinarians.

The inclusion criteria for these participants were healthy adults who had owned a dog for at least two years prior to the interview. The exclusion criterion for this group was losing a pet within the two years preceding contact with the researchers, even if another pet remained in the household. We used a period of two years living with a live dog and without losing any pet in this period as an inclusion criterion because the grief reactions for loss of a companion animal can last up to two years [16].

For both groups, we excluded people who owned a dog for business purposes (with pedigrees from the Israel Dogs Association). The reason for this decision is that dogs raised for profit often live outside the owners' home, in special kennels, sometimes even tended by special staff. In such cases, the effect of pet loss on their owner’s QOL would be different from those who live with their owners who also tend to them. We included in this study only female participants because in a pilot study [26] we saw that that the proportion of male owners in the study sample was small (5 men to 25 women), and those who participated in the study did it only with their wives.

The approval of the Human Subjects Research Committee of the Health Sciences Faculty at Ben-Gurion University of the Negev, Israel, was received for this study. All persons gave their informed consent prior to their inclusion in the study.

### Sample size

The calculation of sample size was performed using a significance level α = 0.05 and power π = 0.8 for QOL changes with standard deviation σ = 18.2 and significant difference 7.2. The calculation was performed with appropriate formulas using statistical calculation programs PS (version 2.1.31) [27]. The calculation was based on preliminary population norms for WHOQOL-BREF questionnaire [28]. All four parts of the questionnaire (Physical health, Psychological well-being, Social relationship, and Environmental domains) have similar means. We performed the calculation using the mean and standard deviation of the Social relationship part of the questionnaire (mean 71.5; *SD* 18.2) because the standard deviation for this part is biggest, and that provides a maximal sample size. Using this calculation, we obtained the minimal sample size of 101 participants for each one of two research groups (current and bereaved owners).

### Instruments

1. *Demographic variables* were measured using a questionnaire containing 12 standard socio-demographic questions that is a part of Israel Center for Disease Control (ICDC) Health Questionnaire (2004).
2. *Quality of life* was assessed by WHOQOL-BREF questionnaire adapted for Israel which contains 26 items. The instrument is divided into four major domains of total QOL—*Physical*, *Psychological*, *Relationship and Environmental*. The Physical domain of QOL represents the functional status and disability, sleeping and working conditions of the participants. The *Psychological* domain of QOL checks the overall enjoyment of the person from life, such as positive feelings, negative feelings, and self-esteem. The *Relationship* domain of QOL checks the person relationship with others, friendship or support he receives from people around him. The *Environmental* domain of QOL checks the financial conditions of the person, such as financial resources, home environment, freedom, physical safety and security [29]. The reliability analysis for the QOL questionnaire found that the obtained Cronbach’s α for the whole questionnaire was 0.87. For the part of the questionnaire related to *Physical* QOL—Cronbach’s α = 0.79, for *Psychological* QOL—Cronbach’s α = 0.56, for the *Relationship* QOL—Cronbach’s α = 0.66 and for the *Environmental* QOL—Cronbach’s α = 0.72.
3. *Stress* was measured using two scales: The Perceived Stress Scale (PSS) [30] and Somatic Complaints scale (SC) [31]. Using these scales, The *Total stress* score was calculated as the sum of all answers on the PSS and SC scales combined. This score represents the total feeling of stress one feels in the current situation. The reliability analysis for PSS: Cronbach’s α = 0.90; for the SC measure: Cronbach’s α = 0.85.
4. *Health behaviors*—we compiled this measure by combining two short instruments. The first was used recently in a study on physicians' emotional well-being and professional functioning conducted at Ben-Gurion University [32]. The second was the ICDC Health Questionnaire (2004). The health behaviors instrument assessed physical activity, smoking status, and weight status.
5. *Social support*—was measured using the 12-item MSPSS (Multidimensional Scale of Perceived Social Support) [33]. This is a self-report questionnaire that assesses the subjective feelings of a person about the social support obtained from three sources—family, friends, and significant other. The reliability analysis for the Social Support Scale found that Cronbach’s α for the entire scale was 0.90. For the part of the scale related to *support from family* we found Cronbach’s α = 0.93; for the part of the scale related to *support from friends*, Cronbach’s α = 0.94; for the part of the scale related to *support from significant other*, Cronbach's α = 0.87.

### Statistical analysis

A three-stage analysis of the data was performed. First we performed descriptive statistics, calculating means and standard deviations for the quantitative variables with a normal distribution (e.g., age). For quantitative variables with non-normal distribution the median and range are presented (e.g., Children living with parents, Living density etc.). For qualitative variables the distributions are presented (e.g., family status, financial status, occupation). The correlation between the four QOL domains was checked using a Spearman correlation coefficient.

Next, univariate analysis of possible relations between the dependent variables and each one of the independent variables was performed. Quantitative and categorical variables were compared by independent group *t* tests (or Mann-Whitney test if a quantitative variable was not normally distributed). Chi-square tests were used to compare two qualitative variables (or Exact Fisher test as appropriate).

At the third stage, multivariate regression models were constructed. Generalized linear model (GLM) for a Gaussian distribution family was used. Dependent variables that did not show a normal distribution (*Relationship* and *Environmental* domains of QOL) were log transformed, and the transformed value used as an independent variable for appropriate regressions. We included independent variables that were significant in univariate analyses at 0.1 significance level for at least one of the owners groups, and variables that were perceived to be important factors on a theoretical basis. The same list of variables was used for both groups of owners. SPSS14 software was used for analysis and processing of all databases. *P* values of ≤ 0.05 were considered to be of statistical significance.

## Results

We obtained 110 completed questionnaires of current owners, and 103 questionnaires of bereaved owners, all women. Socio-demographic and health behavioral variables of the study sample for current and bereaved owners are presented in Table 1.

**Table 1:** Socio-demographic and health behavioral variables of dog owners, by groups.

**Variable**	**Category**	**Current owners** *N* = 110	**Bereaved owners** *N* = 103	***P***
Religious status, *n* (%)	Secular	86 (78.9)	90 (87.4)	.142
	Traditional	23 (21.1)	13 (12.6)	
Family status*, *n* (%)	Never married	43 (39.4)	13 (12.6)	<.001
	Married	52 (47.7)	66 (64.1)	
	Boy/girlfriend	9 (8.3)	12 (11.7)	
	Single	5 (4.6)	12 (11.7)	
Children*, *n* (%)	Yes	58 (52.7)	28 (27.5)	<.001
	No	52 (47.3)	74 (72.5)	
Occupation *, *n* (%)	Employed	77 (70)	79 (77.4)	.007
	Student	26 (23.6)	8 (7.8)	
	Unemployed	7 (6.3)	15 (14.8)	
Financial status, *n* (%)	Good	51 (47.7)	54 (53.5)	.409
	Average	56 (52.3)	47 (46.6)	
Age*, *n* (%)	< than 40 y. old	66 (60.6)	41 (40.2)	.004
	> than 40 y. old	43 (39.4)	61 (59.8)	
Years of education; median (range)		15 (4–24)	16 (1–28)	.446
Children living with parents; median (range)		0 (0–4)	1 (0–4)	.177
Living density; median (range)		0.7 (0.2–1.5)	0.8 (0.3–1.7)	.482
Sports practice, *n* (%)	Not at all	16 (15.1)	18 (18.8)	.431
	Less than once a month	9 (8.5)	8 (8.3)	
	Some times in a month	15 (14.2)	5 (5.2)	
	Once a week	11 (10.4)	12 (12.5)	
	2–4 times a week	40 (37.7)	40 (41.7)	
	Everyday	15 (14.2)	13 (13.5)	
Sports activities in previous two weeks, *n* (%)	Not changed	70 (69.3)	52 (54.2)	.169
	A little less	31 (46.8)	44 (45.9)	
Smoking, *n* (%)	Not at all	82 (75.2)	80 (77.7)	.914
	Smoking	27 (24.7)	23 (22.3)	
Weight, *n* (%)	Not normal	28 (25.7)	34 (34)	.123
	Normal	81 (74.3)	66 (66)	

*significant differences by Chi-square test

The data presented in Table 1 indicates that participants in both groups are mostly secular, married, employed and of good financial status. For the most part they were non-smokers, of normal weight, who engaged in sports 2–4 times a week.

The two groups differed significantly in several demographic variables. *Age*—on the average, the bereaved owners group was older than the current owners group. *Family status* (χ^2^ = 20.9, *p* < 0.01), with more never married among current dog owners; and *occupation* (χ^2^ = 12.2, *p* = 0.01) with more students among current owners. More bereaved owners were parents (χ^2^ = 14.0, *p* < 0.01), but there was no difference between the groups in the *number of children living with parents* (*p* = 0.18).

Dog’s sex did not differ between current and bereaved owners. In both groups approximately 50% of the dogs were male (51.9% in current owners’ group and 45.1% in bereaved owners group). Illness was the main reason for death (89.3% of the dead dogs); 9.3% of the dogs died because of an accident. The median length of ownership of the dog for the bereaved group was 14 years (range 1–25). The median length of ownership of the dog for the current owners’ group was 6.3 years (range 2–18). The length of ownership of the dog was higher for bereaved owners (Mann-Whitney test, Z = -8.1, *p* < 0.01). No other differences in socio-economic, health behavior and dog-related variables were found.

The *Total stress* variable had a normal distribution. There was a significant difference (*p* = 0.01) in *Total stress* between current and bereaved owners (Table 2). All mean values of QOL domains among bereaved owners (except *Environmental* QOL) were significantly lower than the mean values among current owners. The statistical description of each one of the QOL domains for each group of owners is shown in Table 2.

**Table 2:** Statistical description of stress and QOL variables, by groups .*

**Variable**	**Current owners**	**Bereaved owners**	***P***
	Mean ± SD/ median (range)	Mean ± SD/ median (range)	
Total stress	55.9 ± 12.3	61.0 ± 15.2	.01
Physical QOL*	28.3 ± 3.8	26.2 ± 4.8	<.01
Psychological QOL*	25.9 ± 3.1	24.6 ± 3.8	.02
Relationship QOL**	12 (4–15)	11 (6–15)	.02
Environmental QOL**	27 (14–35)	27 (15–35)	.64

*Analysis performed using independent variable Student’s t-test (Normally distributed)

**Analysis performed using Mann-Whitney test (Poisson distributed)

Table 3 shows the distribution of the three *social support* components (support from family, support from friends and support from significant other). Both bereaved and current owners mostly showed high levels of social support obtained from friends, family and significant others.

**Table 3:** Statistical description of social support components, by groups.

**Variable** *	**Current owners**	**Bereaved owners**	***P***
	Median (range)	Median (range)	
Support from family	25 (4–28)	25 (4–28)	.74
Support from friends	26 (7–28)	23.5 (4–28)	<.01
Support from significant other	28 (11–28)	27 (12–28)	.22

*Analyses performed using Mann-Whitney test.

The two groups of owners differed significantly in support from friends (current owners reported higher levels of support, Mann-Whitney test, Z = -3.19, *p* < 0.01). The groups did not differ in the two other components of social support (*support from families* and *support from significant other*).

Weak but significant correlations were found among the three support variables—*support from friends*, *support from families*, and *support from significant other* in both groups of owners. All correlations were significant at the 0.01 level. Support variables were not normally distributed, so Spearman’s correlation was performed (Table 4). All three of the support variables were introduced into the final models.

**Table 4:** Correlation coefficients (Spearman correlation) between support variables, by groups.

**Variable**	**Current owners**		**Bereaved owners**	
	Support from friends	Support from significant other	Support from friends	Support from significant other
Support from family	.38	.51	.49	.70
Support from friends	1	.55	1	.49

Correlations are significant at 0.01 level

Four QOL domains were correlated significantly at 0.01 level (Table 5). The correlation between QOL domains was higher in bereaved owners. All correlations between QOL domains were weak to moderate (r_s_ 0.29–0.79). The highest correlation was found between *Physical* and *Psychological* domains of QOL in bereaved owners. For each of the four QOL domains we performed multivariate linear regression analyses, separately for current and bereaved owners.

**Table 5:** Correlation coefficients (Spearman correlation) between QOL domains.

**Variable** *	**Current owners**				**Bereaved owners**			
	Physical QOL	Psychological QOL	Relation-ships QOL	Environmental QOL	Physical QOL	Psycho-logical QOL	Relationships QOL	Environmental QOL
Physical QOL	1	.29	.26	.45	1	.79	.47	.63
Psychological QOL		1	.32	.43		1	.62	.69
Relationships QOL			1	.19			1	.49

*Correlations are significant at 0.01 level

*The Relationship* and *Environmental* domains of QOL were not normally distributed. The logarithmic transformation was used for normalization of the above-mentioned dependent variables. The logarithmic form of these variables was used in the regression models. Univariate associations between QOL and each one of the social support variables are presented in Table 6.

**Table 6:** Significance of univariate associations among the three QOL domains and the Social support components, by groups.

**Variable** *	**Current owners**								**Bereaved owners**							
	Physical QOL		Psycho-logical QOL		Relation-ships QOL		Environmental QOL		Physical QOL		Psycho-logical QOL		Relation-ships QOL		Environmental QOL	
	r	P	r	P	r	P	r	P	r	P	r	P	r	P	r	P
Support from family	-.17	.08	-.01	.91	.24	.02	.25	.01	.39	<.01	.43	<.01	.56	<.01	.37	<.01
Support from friends	.23	.02	0.3	<.01	0.4	<.01	.19	.04	.30	<.01	.45	<.01	.42	<.01	.22	.08
Support from significant other	.08	.41	0.2	.12	0.4	<.01	.15	.13	.39	<.01	.51	<.01	.59	<.01	.47	<.01

*Analysis performed using Spearman correlation

### 1. The Physical domain of quality of life

For each of the two groups we analyzed by multiple linear regressions the associations between the *Physical* domain of QOL and the independent variables that were found significant in univariate analyses (Table 7). For current dog owners the only factor that predicts the *Physical* domain of QOL is *total stress* (negative association; more stress reduces the *Physical* domain of QOL). The proposed regression explains 17.6% of total variance for the *Physical* QOL dependent variable.

**Table 7:** Results of multivariate analyses of factors related to the Physical domain of QOL, by groups.

**Variable**	**Current owners**					**Bereaved owners**				
	B	SE	Beta	P	95%-CI	B	SE	Beta	P	95%-CI
Constant	34.1	2.9		<.01	28.4; 39.8	35.8	2.1		<.01	31.5; 40.1
Support from friends	0.1	0.1	0.1	.39	-0.1; 0.2	0.2	0.1	0.2	<.01	0.1; 0.3
Total stress	-0.1	0.03	-0.4	<.01	-0.2; -0.1	-0.2	0.02	-0.7	<.01	-0.3; -0.17
Adjusted R^2^	0.18					0.53				

Among bereaved owners the factors that are significantly correlated with the *Physical* domain of QOL are *support from friends* (a positive correlation; higher support from friends increases the *Physical* QOL) and *total stress* (a negative correlation; higher stress levels reduce *Physical* QOL), and is the strongest predictor for this regression. The proposed regression explains 53.4% of total variance for the *Physical* domain of QOL as a dependent variable. Overall, for both groups the regression explained 40.0% of total variance for the *Physical* domain of QOL.

### 2. The Psychological domain of quality of life

The associations between the *Psychological* domain of QOL and independent variables are presented in Table 8. For current owners *age* predicts the *Psychological* domain of QOL (being older than 40 negatively correlates with this parameter and is a risk factor for the *Psychological* domain of QOL). The strongest predictor of *Psychological* QOL in this group is *total stress* that negatively correlates with the *Psychological* QOL. The proposed regression explains 17.4% of total variance for the *Psychological* QOL dependent variable.

**Table 8:** Results of multivariate analysis of factors related to the Psychological domain of QOL, by groups.

**Variable**	**Current owners**					**Bereaved owners**				
	B	SE	Beta	P	95%-CI	B	SE	Beta	P	95%-CI
Constant	28.7	2.9		<.01	22.7; 34.6	26.1	2.6		<.01	20.9; 31.2
Age (grouped)	-1.6	0.6	-0.3	.01	-2.9; -0.4	-1.3	0.6	-0.2	.03	-2.5; -0.2
Support from friends	0.1	0.1	0.2	.16	-0.4; 0.3	0.2	0.1	0.2	<.01	0.1; 0.3
Support from significant other	-0.01	0.1	-0.01	.95	-0.2; 0.2	0.2	0.1	0.2	<.01	0.1; 0.4
Total stress	-0.1	0.02	-0.3	<.01	-0.1; -0.03	-0.2	0.02	-0.6	<.01	-0.2; -0.1
Adjusted R^2^	0.17					0.60				

For bereaved owners the *Psychological* domain of QOL correlates with *age* (being older than 40 negatively correlates with *Psychological* domain of QOL and is a risk factor for the *Psychological* domain of QOL); *support from friends* and *support from significant other* positively correlate with *Psychological* domain of QOL; and *total stress* is a risk factor for and the strongest predictor of *Psychological* QOL. The proposed regression explains 59.7% of total variance for *Psychological* QOL. Overall, for both groups the regression explained 41.5% of total variance for the *Psychological* QOL dependent variable.

### 3. The Relationship domain of quality of life

The associations between the *Relationship* domain of QOL and the independent variables are shown in Table 9.

**Table 9:** Results of multivariate analysis of factors of the Relationship domain of QOL, by groups.

**Variable**	**Current owners**					**Bereaved owners**				
	B	SE	Beta	P	95%-CI	B	SE	Beta	P	95%-CI
Constant	7.5	1.9		<.01	3.6; 11.3	2.8	1.6		. 07	-0.2; 5.9
Support from family	-0.1	0.04	-0.2	.03	-0.2; -0.01	0.1	0.04	0.2	.05	0.002; 0.2
Support from friends	0.1	0.05	0.3	.02	0.02; 0.2	0.1	0.03	0.2	.03	0.01; 0.1
Support from significant other	0.2	0.1	0.3	.02	0.02; 0.2	0.2	0.1	0.4	<.01	-0.1; 0.3
Total stress	-0.1	0.02	-0.3	<.01	-0.1; -0.02	0.01	0.01	-0.04	.62	-0.3; 0.02
Adjusted R^2^	0.36					0.47				

For current owners, the *Relationship* domain of QOL, *total stress* and all three components of support—*support from family*, *support from friends* and *support from significant other*, were significant. Two components—*support from friends* and *support from significant other—*are positively correlated to *Relationship* QOL. *Support from significant other* is the strongest predictor for this model. *Support from family* negatively correlates with *Relationship* domain of QOL. *Total stress* negatively correlates with *Relationship* QOL in this group, thus higher stress levels reduce the *Relationship* QOL. The proposed regression explains 33.5% of total variance for *Relationship* QOL dependent variable. For bereaved owners, three support factors are important for the *Relationship* domain of QOL—*support from family*, *support friends* and *support from significant other*. All of them positively correlate with *Relationship* domain of QOL. The proposed regression explains 47.0% of total variance for *Relationship* domain of QOL dependent variable. Overall, for both groups the regression explained 40.8% of total variance for *Relationship* domain of QOL dependent variable.

### 4. The Environmental domain of quality of life

The two groups of owners were not significantly different in the *Environmental* domain of QOL (Table 2). For both groups the *Environmental* domain of QOL negatively correlates with the *financial status* of owners with a significance level of *p* < 0.01. Being of average financial status is a risk factor for the *Environmental* domain of QOL. For current owners *support from friends* was also significantly correlated with the *Environmental* domain of QOL (*p* = 0.04) thus higher support levels raise the *Environmental* domain of QOL. The proposed regression explains 13.9% of total variance for the *Environmental* domain of QOL dependent variable for current owners and 42.4% for bereaved owners.

A summary of the variables that are significant predictors of QOL domains is shown in Table 10. For different domains of QOL, three major variable groups were significant—demographic variables (*age* and *financial status*), social support and stress.

**Table 10:** Variables that are predictors of different QOL domains, by groups.

**Dependent variable**	**Current owners**	**Bereaved owners**
Physical QOL	Total stress (-)	Total stress (-)
		Support from friends (+)
Psychological QOL	Total stress (-)	Total stress (-)
	Age (-)	Age (-)
		Support from friends (+)
		Support from significant other (+)
Relationship QOL	Total stress (-)	Support from family (+)
	Support from family (-)	Support from friends (+)
	Support from friends (+)	Support from significant other (+)
	Support from significant other (+)	
Environmental QOL	Financial status (-)	Financial status (-)
	Support from friends (+)	

## Discussion

In our study we investigated differences in QOL between current owners of live dogs and owners who lost their dog (bereaved owners). The levels of QOL in three of the four QOL domains (*Physical*, *Psychological*, and *Relationship*) of current owners were significantly better than the levels among bereaved owners. These findings reflect the negative contribution to well-being of losing a dog, and support previous studies that concluded that dogs confer psychological and physical benefits on their owners [5, 34–35]. It should be noted, however, that in this study we can infer the positive role of owning a dog only indirectly, because we did not include a group of participants without dogs, as was done in previous studies.

Stress was significantly associated with *Physical*, *Psychological*, and *Relationship* domains of owners’ QOL. It appears that the death of a dog is a stressful life event, most probably because dog owners have a great level of attachment to their companion pets [14, 36]. The loss of an attachment figure provokes a stress reaction [21, 25].

We did not find an effect of health behaviors on owners’ QOL. For the four-week period during which we met with bereaved owners, the effect of interrupting the sports activities could not be determined.

Quality of life of owners was found to be positively associated with social support. Social support is important in times of trouble and disaster, when one needs more support than usual [37–38]. One such case may be the death of a dog, an event which increases external stress and the need for love [20]. Moreover, the deceased dog itself may have been a source of implicit support [39]. The negative association between social support and stress found in this study correlates with such effects found in the case of the death of a human being [22]. In the case of mourning for a person, social support is very common and expected, but when a pet dies people do not always grasp the depth of the bereaved owner’s sadness. Lack of social support in the case of death of a companion animal may strongly affect owner’s grief reactions. In our previous study [26] we saw that close family members are not always able to provide support for bereaved dog’s owners. This is reflected in the study reported here, where support from family is related only to the Relationship domain of QOL both for current and bereaved owners, while the positive role of support from friends and from a significant other is related directly and indirectly to all four domains of QOL—Physical, Psychological, Relationship, and Environmental and can improve the QOL both of current and of bereaved owners. It has to be noticed that most of the families understood the problems of bereaved owners (support from family is positively correlated with the Relationship QOL), while this factor is negligible in the current owners group. Social support is the only factor we found to be related to all QOL domains, that proves the extremely important role of social support in people’s lives.

Several studies show that society does not always acknowledge the significance of pet bereavement [40–41]. In the USA, some veterinary hospitals organize support groups for adults, especially for elderly people, to help them accept the loss [42–43], and hotlines are available around the clock for those who cannot cope with their feelings. Such practices are consistent with some authors' opinions that showing appropriate sentiments toward grieving pet owners (calling the next day after euthanasia, writing condolence letters) is part of the vet’s obligations [44–45].

*Age* in our study was related directly only to current owner’s QOL. In a previous study it was shown that women over 40 who were pet owners had worse mood swings than their counterparts who did not own pets [46], so we expected to see benefits in QOL parameters for bereaved female owners older than 40. We did not see this effect. However, we know from previous studies that a death of a dog is more stressful for elderly people [15, 47]. In our study we did not see this effect because the maximal age of owners was 65, so we had no elderly participants.

It is notable that in all the regression analyses in this study the total variance of the models performed for bereaved owners was much higher than for the current owners’ group (all adjusted *R* ^2^ were higher in the case of bereaved owners). In other words, the regression models explained the levels of QOL of bereaved owners much better than those of current owners. It seems that for current owners the dogs are only one of a large number of factors that affect their everyday stress and QOL. In the case of bereaved owners the loss of the dog may be one of the most important things that affect well-being, at least at the short term, close to the dog's death.

## Limitations of the study

### The study has several limitations.

First, the most important limitation of this study was that the sample included only those owners who agreed to participate. It is probable that the owners who did not agree to participate in the study would provide a different picture of emotions and behaviors.

Second, we included only female owners. Some authors found gender differences in health and QOL, as a result of death of a spouse [48, 49], thus it can be assumed that some differences could be also found in a case of death of a dog. However, in a study by Rajaram et al. (1993), no gender effects were found on grief reactions or degree of grief following animal death. Future investigations of such gender differences seem to be necessary [50].

Additionally, there could be a potential bias in subject selection in both groups. For the bereaved group, relying on veterinary records necessarily negates the opportunity to recruit subjects who did not take the dog to the vet for euthanasia. This would include dogs killed instantly in traffic or other accidents and dogs dying at home of old age. It is possible that these subjects invest less on veterinary care and are possibly less emotionally attached to the dog. For the non-bereaved group similar bias may be introduced by relying on veterinary referrals.

Despite these limitations our study, which focused on healthy participants, explained a wide range of current and bereaved owner’s reactions and effects of different dog-related factors on owners QOL.
